# Abnormal handling mechanism and improvement measures of optical DC current transformer in smart grid environment

**DOI:** 10.7717/peerj-cs.1132

**Published:** 2022-11-01

**Authors:** Shihao Zhou, Hansong Tang, Benren Pan, Wei Zhang

**Affiliations:** 1 Electric Power Research Institute of State Grid Jiangxi Electric Power Co., Ltd, Nanchang, Jiangxi, China; 2 Jiangsu LingChuang Electric Automation Co., Ltd, Zhenjiang, Jiangsu, China

**Keywords:** FOCT, Modulation circuit, Bessel function, Compensation circuit, Abnormal handling mechanism, Transient test system

## Abstract

An optical DC current transformer anomaly handling mechanism is proposed to address the problem that the conventional DC current transformer anomaly handling mechanism cannot compensate for the defect of capacitor anomaly blocking. First, the implementation principle, modulation loop, demodulation method and its anomaly warning mechanism of the sine-wave modulated all-fibre-optic current transformer (FOCT) are investigated, and the effects of light source intensity and modulation voltage on current decoding are explained. The modulation loop is then simulated and modelled and a FOCT anomaly handling mechanism is proposed based on the Bessel function with real-time dynamic current compensation for small changes in modulation depth. Finally, an integrated dynamic test system for DC current transformers and DC protection is designed, and the actual system operation and fault model is established using the RTDS simulation system. The experiments demonstrate that the proposed FOCT anomaly handling and improvement measures can effectively improve the transient performance of FOCT, and at the same time provide a complete set of testing means for the engineering application and later upgrade and replacement of FOCT.

## Introduction

In the process of increasing the transmission capacity and voltage level of the power system, the traditional DC transformer is limited by its own sensing mechanism and presents problems such as easy distortion of the measurement signal under high currents, so the new current transformer represented by the optical current transformer has great potential for development.

With the continuous transformation of traditional power grids into smart grid, a high proportion of new energy sources represented by wind power and energy storage and a large number of power electronics are connected to the grid, inducing new problems in grid operation such as protection malfunctions and phase change abnormalities, so improving the abnormal handling mechanism of power electronics has become an important research topic.

Yalong River DC undertakes the work of clean energy transmission from Yalong River Hydropower Station after putting into system operation, starting from Yalong River Converter Station in Liangshan Yi Autonomous Prefecture of Sichuan Province and ending at Poyang Lake Converter Station in Fuzhou City of Jiangxi Province. Among them, the DC electronic current transformer of Poyang Lake converter station mainly adopts all-fiber optic current transformer has shown abnormal alarm many times in the operation process and led to DC protection false operation. The common all-fiber optic current transformers (Li et al., 2018; Dziuda et al., 2007; Wu & Zhang, 2020; Li et al., 2019b) are divided into sine wave modulation (open-loop modulation) and square wave plus step wave modulation (closed-loop modulation) according to their modulation methods (Wang, Zhang & Pang, 2021; Pang et al., 2020; Wu, Zhang & Chen, 2022). The sine wave modulated full optical fiber current transformer (FOCT) used in Poyang Lake converter station is the mainstream DC electronic type current transformer currently operating in UHV DC converter station, and its reliability is directly related to the safety and reliability of DC transmission system, so its fault mechanism and its abnormality alarm mechanism are also receiving increasing attention (Temkina, Medvedev & Mayzel, 2020; Zhao et al., 2022; Qi et al., 2020; Bashir Ahmed, 2019; Chen, Xu & Wang, 2020). In Hu et al. (2019), the long-term temperature error due to Verdet’s constant was compensated by selecting a suitable initial phase delay of the waveplate in order to optimize the FOCT accuracy, but the waveplate accuracy was difficult to control due to the different absorption coefficients of unusual and unusual light in the ${\lambda/4}$ waveplate. In Zhao, Shi & Sun (2020), an optical fibre temperature sensing scheme based on the temperature birefringence effect of the polarisation-preserving fibre (PMF-TS) was proposed, but the structural defects generated inside the fibre during the drawing process of the polarisation-preserving fibre can cause degradation of the polarisation-preserving performance. The existing research on optical current transformers mainly focuses on the abnormal manifestations of optical DC electronic current transformers, and there is a lack of research on the causes of faults and alarm mechanisms because the FOCTs are all foreign imported equipment.

In this article, based on the FOCT accident analysis of Poyang Lake converter station, the current performance characteristics of the FOCT modulation circuit when it is abnormal and the defects of its abnormal warning mechanism are studied from the perspective of FOCT modulation principle through modelling simulation and physical simulation. The innovations of this study, compared to the traditional methods of improving DC current transformers, are.

1. The proposed improved mechanism for abnormality handling of FOCT solves the problem of abnormal output currents caused by sudden changes in the modulation voltage of the modulation loop in the traditional method and the inability to warn by improving its existing abnormality warning mechanism to suit the application requirements of DC protection.
2. The integrated dynamic test platform of DC current transformer and DC protection is built, and the ground engineering practice provides a complete set of testing means for the engineering application and later upgrade and replacement of FOCT.

## Analysis of the cause of foct accident in poyang lake converter station

**Figure 1:**
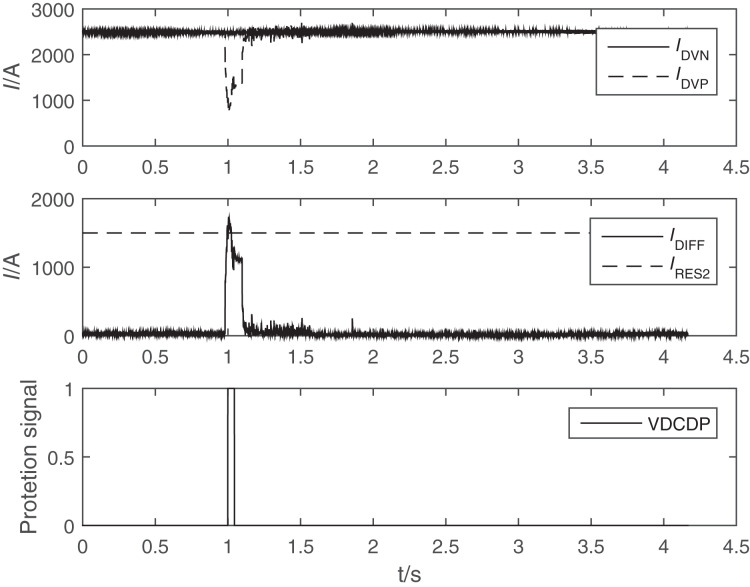
Waveform of differential protection outlet of valve block connecting line of Poyang Lake converter station. I_DVN_ and I_DVP_ are the two measuring points of valve converter differential protection (VDCDP). I_DVP_ is fault FOCT current. I_DIFF_ is the differential current of converter differential protection, I_RES_ is the restraint current of converter differential protection.

At 11:37 on August 22, 2021, the FOCT sampling output of pole II low end valve group in Poyang Lake converter station is abnormal. As shown in Fig. 1, the differential current of pole 2 low-end converter meets the action setting of converter differential protection II section, and the delay is 5 ms to protect the outlet. The differential current of pole 2 valve group connection line meets the action value of valve group connection line differential protection II, and the delay time is 6 ms to protect the export. I_DVN_ and I_DVP_ in Fig. 1 are the two measuring points of valve converter differential protection (VDCDP). I_DVP_ is fault FOCT. I_DIff_ is the differential current of converter differential protection, I_RES_ is the restraint current of converter differential protection.

Upon inspection, it was found that the modulator shunt compensation capacitor within the CMB of the pole 2 low-end FOCT phase modulator had a false solder.

**Figure 2:**
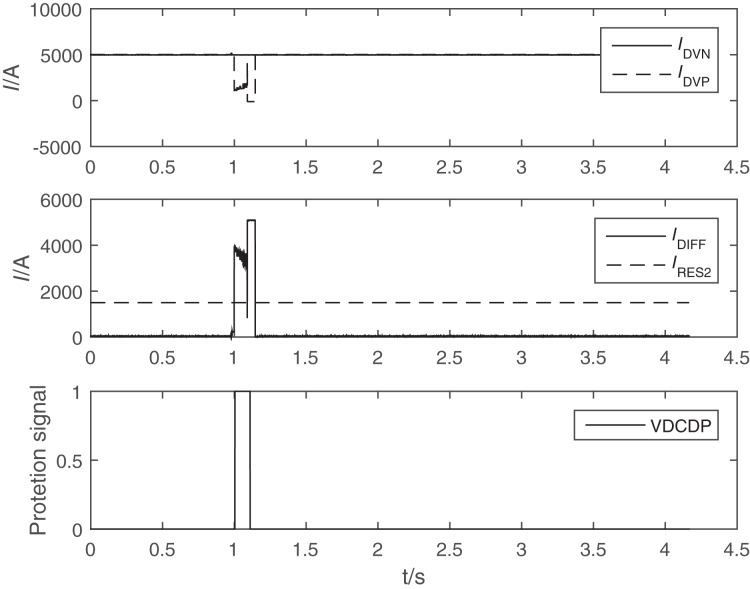
Waveform of modulation circuit parallel capacitance unstable connectionof FOCT in laboratory. I_DVN_ and I_DVP_ are the two measuring points of valve converter differential protection (VDCDP). I_DVP_ is fault FOCT current. I_DIFF_ is the differential current of converter differential protection, I_RES_ is the restraint current of converter differential protection.

In this article, we use the same type of FOCT in the laboratory, simulate the field 400 m long distance modulation cable connection test FOCT and electronic unit, use the current source to add 5,000 A DC current to the test CT, the output test results of the electronic unit is shown in Fig. 2.

In the test process, the fault situation in the field was reproduced by frequent connection and disconnection of the shunt capacitor of the modulation loop. The output current of the electronic unit was frequently cleared due to frequent setting of the quality position to 1, and occasionally the output was abnormal and the current quality position was not set to 1.

Through field accidents and laboratory tests, it can be determined that the existing design of the all-fiber-optic current transformer (FOCT) in the modulation circuit shunt capacitor connected and disconnected frequently for a short period of time will have a low probability of not destroying the modulation self-locking logic, and the alarm message cannot be triggered, resulting in an abnormal output of the FOCT sampling value when the modulation circuit is abnormal and its related blocking protection error marker is not set on, leading to DC differential protection misoperation. This can lead to the accident that the DC differential protection is misactivated to block the DC system.

## Modulation principle of sine wave modulated all fiber optic current transformer (foct) and its alarm mechanism

As an interactive system, the smart grid requires power electronics to respond quickly to changes in it while ensuring system safety. At present, the development of existing power electronics devices focuses on the implementation of basic functions and performance improvement, with little consideration given to intelligence and support for the upper layers. Therefore, improving the abnormal processing of power electronic devices in grid operation can effectively improve the quality of electrical energy, while also enhancing the transmission efficiency of the grid system and realizing good interaction between users and power supply enterprises, thus realizing the intelligent and automated development of the grid.

### Implementation principle of all-fiber optic current transformer

All-FOCT generally use two beams of polarized light that are always transmitted in two orthogonal modes on the same sensing fiber, and in the sensing fiber, the two beams of circularly polarized light are transmitted at different speeds due to the Faraday magneto-optical effect of the magnetic field generated by the current in the wire, resulting in the Faraday phase difference (Karabulut et al., 2019; Wu et al., 2021; Mihailovic & Petricevic, 2021). Therefore, the two beams carrying the Faraday effect phase information will interfere at the starter and then be coupled by the 3 dB coupler into the photodetector, and the interferometric light intensity information arriving at the photodetector is equationed as

(1) $${P_{{\mathop{\rm int}} }} = 0.5E_x^2 \cdot [1 + \cos (4NVI)]$$

where *E*_*x*_ is the light intensity of the light source, *N* is the number of turns of the sensing fiber, *V* is the Verdet constant of the sensing fiber, and *I* is the value of the current flowing in the current-carrying conductor.

As can be seen from Eq. (1), the output light intensity does not reflect the sign of the Faraday phase shift and the first order derivative of the cosine function at zero phase is zero, making the current output signal does not have the two disadvantages of distinguishing the current direction and low detection sensitivity. Therefore, it is necessary to phase modulate the two beams of line polarized light by a phase modulator to shift the system operating point to other positions, so as to achieve the full range of accurate measurement in both directions.

### Phase modulation method of FOCT

The FOCT in Poyang Lake converter station all adopt the sine wave modulation method of piezoelectric ceramic PZT modulator, and the electronic unit applies a sine wave voltage signal to the PZT. The voltage causes the change of refractive index of the material inside the modulator, and the propagation speed of the light passing through changes, thus causing the phase change of the light. In general, the phase of the light is linearly related to the applied modulating voltage, so the relationship between the phase and the modulating voltage under the action of the sinusoidal modulating voltage is shown in Eq. (2).

(2) $$\varphi = \displaystyle{\pi \over \lambda }rn_1^3\displaystyle{L \over D}U = kU$$

In Eq. (2), D is the distance between the two electrodes, L is the electrode length, n1 is the refractive index of the optical waveguide, r is the photoelectric coefficient of the waveguide material, U is the modulation voltage, and k is the modulation coefficient of PZT.

At this time the light intensity expression is.

(3) $${P_{{\mathop{\rm int}} }} = 0.5E_x^2 \cdot [1 + \cos (4NVI + \varphi )]$$

For the same beam of polarized light polarized beam in the polarization-preserving fiber delay line and sensing head transmission, after τ time when returned to the phase modulator, then the beam of polarized light before and after the combined effect of phase modulation that modulation depth function is $\varphi_{\rm mod}(t)= \varphi{(t+\tau)}- \varphi(t)$. The modulation function in case of sinusoidal modulation is Eq. (4).

(4) $$U(\rm t) = {U_m}{\rm sin}(\omega t)$$

In Eq. (4), *U*_*m*_ and *ω* are the amplitude and angular frequency of the modulated voltage reflected in the phase modulation can be obtained as follows

(5) $$\varphi_{\rm mod}(t) = \varphi \left( t \right) - \varphi \left( {t - \tau } \right) = 2k{U_{\rm m}}\sin \left( {\displaystyle{{\omega \tau } \over 2}} \right)\cos (\omega t)$$

The modulation coefficient *k*, angular frequency *ω*, and delay time *τ* in Eq. (5) are all designed parameters for fixed values, so the modulation depth $\varphi_{\rm mod}={2kU_{\rm m}{\rm sin}(0.5\omega \tau)}$ of the modulation loop can be set, and the modulation depth remains linearly related to the modulation voltage.

Substituting Eq. (5) into Eq. (3) yields the interferometric light intensity information equation.

(6) $${P_{{\mathop{\rm int}} }}(t) = \displaystyle{{E_x^2} \over 2} \cdot \left( {1 + \cos \left( {4NVI + \varphi _{\rm mod}\cos \left( {\omega t} \right)} \right)} \right)$$

### Demodulation and anomaly alerting mechanism of FOCT

Since Bessel functions are solved using the idea of a series of solutions, at the same time Bessel functions of different orders are not isolated from each other, but have a certain recursive relationship. The Bessel function also has the typical orthogonality, which can be used to find the magnitude of the hypothetical solution level and coefficients. Therefore the Bessel function is chosen for this study to be solved.

According to Eq. (6), it can be seen that the interference light intensity information is influenced by the light intensity of the light source, the modulation depth and the three factors of the flowing current.

(7) $${P_{{\mathop{\rm int}} 1}} = - E_x^2 \cdot {J_1}\left( {{\rm }{\varphi _{\rm mod }}{\rm }} \right){\rm sin}\left( {4NVI} \right)$$

(8) $${P_{{\mathop{\rm int}} 2}} = - E_x^2 \cdot {J_2}\left( {{\rm }{\varphi _{\rm mod }}{\rm }} \right)\cos \left( {4NVI} \right)$$

(9) $${P_{{\mathop{\rm int}} 4}} = E_x^2 \cdot {J_4}\left( {{\rm }{\varphi _{\rm mod }}{\rm }} \right)\cos \left( {4NVI} \right)$$

where *J*_1_( $\varphi_{\rm mod}$), *J*_2_( $\varphi_{\rm mod}$) and *J*_4_( $\varphi_{\rm mod}$) are the 1st, 2nd, and 4th order Bessel functions corresponding to the modulation depth $\varphi_{\rm mod}$ of the modulation loop, respectively. Since the Faraday phase shift is generally small, the remaining sine value is much larger than the sine value. In order to improve the sensitivity of the solution and eliminate the influence of the light intensity of the light source and the modulation depth, the ratio of *P*_int1_ to *P*_int2_ and the ratio of *P*_int2_ to *P*_int4_ are obtained as follows

(10) $$\displaystyle{{{P_{{\mathop{\rm int}} 1}}} \over {{P_{{\mathop{\rm int}} 2}}}} = \displaystyle{{{J_1}\left( {{\rm }{\varphi _{\rm mod }}{\rm }} \right)} \over {{J_2}\left( {{\rm }{\varphi _{\rm mod }}{\rm }} \right)}}\tan \left( {4NVI} \right)$$

(11) $$\displaystyle{{{P_{{\mathop{\rm int}} 2}}} \over {{P_{{\mathop{\rm int}} 4}}}} = {\rm - }\displaystyle{{{J_2}\left( {{\rm }{\varphi _{\rm mod }}{\rm }} \right)} \over {{J_4}\left( {{\rm }{\varphi _{\rm mod }}{\rm }} \right)}} = {\rm - }{k_{bessel24}}$$

For the convenience of solving the calculation at small angles the angle approximation equation 4*NVI* ≈ tan(4*NVI*) is used. At the same time, according to Eq. (11), it is known that the ratio of the 2nd and 4th harmonics of the interference light intensity information is the ratio of the 2nd and 4th order Bessel functions *k*_bessel24_, and the electronic unit controls the modulation depth $\varphi_{\rm mod}$ by monitoring the value of *k*_bessel24_ closed-loop modulation voltage value, so that *J*_1_( $\varphi_{\rm mod}$) = *J*_2_( $\varphi_{\rm mod}$), thus obtaining the final current solution equation.

(12) $$I \approx \displaystyle{1 \over {4VN}}\displaystyle{{{P_{{\mathop{\rm int}} 1}}} \over {{P_{{\mathop{\rm int}} 2}}}}$$

From the above it is known that in order to ensure the accuracy of current measurement of FOCT. So the phase shift of the interferometric light intensity information of the FOCT transduction current satisfies.

(13) $$\left| {4NVI} \right| < \pi /12$$

As the Faraday phase shift is generally small when working normally, so the remaining sine function is approximated as one, the value of *P*_int2_ response is the product of the light intensity and modulation depth of the 2nd order Bessel function under normal circumstances, its value is not affected by changes in load current, but both the optical circuit or modulation circuit is abnormal will lead to a reduction in the amplitude of *P*_int2_, so the actual engineering is generally used *P*_int2_ as the basis for the judgment of abnormalities, the judgment equation is

(14) $$\left| {{P_{{\mathop{\rm int}} 2}}} \right| < E_x^2 \cdot {J_2}\left( {{\rm }{\varphi _{\rm mod }}{\rm }} \right)\cos \left( {\pi /12} \right)$$

That is, when the absolute value of *P*_int2_ is lower than the maximum measured current causing phase shift (outside the measurement range) FOCT will issue an alarm message as the basis for DC protection blocking.

## Foct compensation capacitor abnormal blocking defect and its improvement measures

Since the electronic unit of FOCT in engineering practice is in the main control room and the phase modulator is usually in the primary site together with the sensing fiber. Therefore, a long cable is required for connection, and the cable is mainly inductive at high-frequency signals, and compensation capacitors need to be added at both ends of the phase modulator to compensate for the phase and amplitude of the modulation voltage at both ends of the PZT (Zhao, Xu & Sun, 2021; Yan et al., 2018).

**Figure 3:**
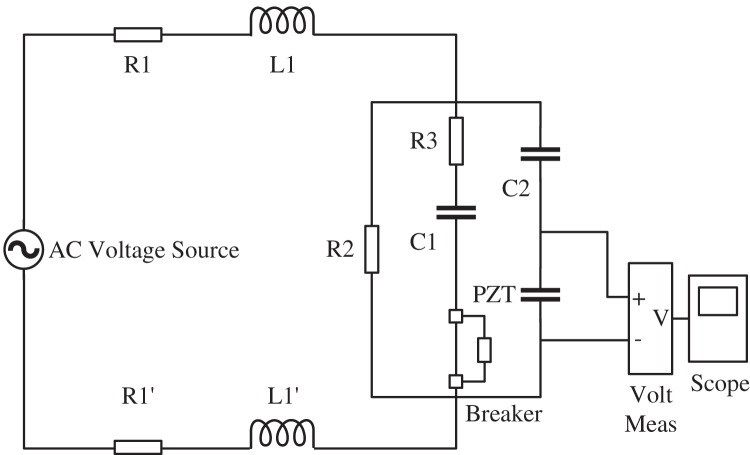
Simulation circuit of FOCT modulation circuitunstable connection. The simulation circuit diagram of the modulation loop is show as the figure. R_1_, L_1_, R_1_′, L_1_′ are the equivalent loop resistance and inductance of 400 m control cable, R_1_ = R_1_′ = 1.65 Ω, L_1_ = L_1_′ = 0.08 mH. The shunt resistance of sampling R_2_ = 1.5 k $\Omega$, the enabling resistance of compensation capacitor R_3_ = 15 $\Omega$, and the compensation capacitor C_1_ = 42 nF.

**Figure 4:**
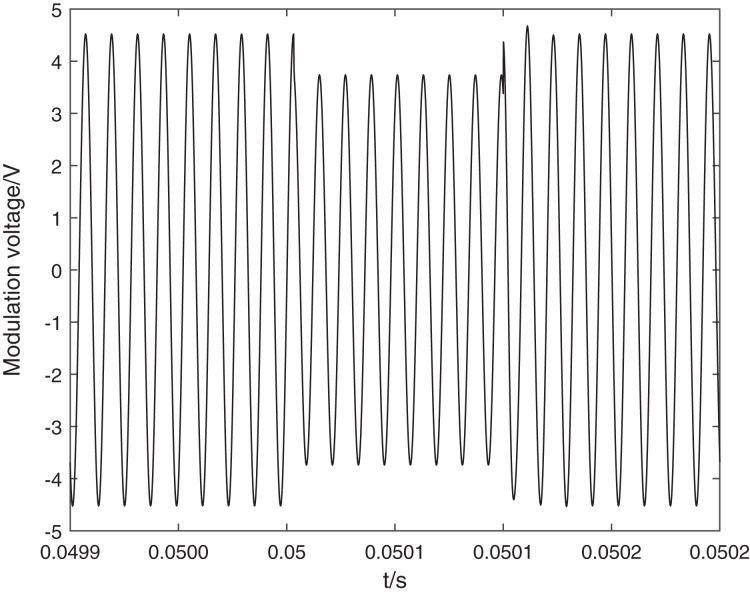
Simulation voltage of FOCT modulation circuit capacitor circuit completely disconnected. The black line indicates the modulation voltage. When the compensation capacitor is falsely connected, it leads to a change in the actual modulation voltage amplitude and is accompanied by a significant transient process.

In this article, we use MATLAB to build the simulation circuit diagram of the modulation loop, as shown in Fig. 3, to simulate the process of compensating the false connection of the capacitor loop, and the simulation results of the voltage output when the FOCT modulation loop capacitor loop is completely disconnected are shown in Fig. 4. In Fig. 3, R_1_, L_1_, R_1_′, L_1_′ are the equivalent loop resistance and inductance of 400 m control cable, R_1_ = R_1_′ = 1.65 $\Omega$, L_1_ = L_1_′ = 0.08 mH. The shunt resistance of sampling R_2_ = 1.5 kΩ, the enabling resistance of compensation capacitor R_3_ = 15 Ω, and the compensation capacitor C_1_ = 42 nF.

The simulation results are shown in Fig. 4. When the compensation capacitor is falsely connected, it leads to a change in the actual modulation voltage amplitude and is accompanied by a significant transient process. Since $\varphi_{\rm mod}$ is linearly related to the modulation voltage eventually makes the modulation depth $\varphi_{\rm mod}$ jump, the actual reduction is related to the contact resistance size, and the relationship between contact resistance and $\varphi_{\rm mod}$ reduction is shown in Table 1.

**Table 1:** Relationship between contact resistance and output voltage reduction.

Contact resistance ( $\Omega$)	$\infty$	30	15	10	5
$\varphi_{\rm mod}$ reduction margin (ε%)	7.9	9.7	6.2	4.4	2.8

**Note:**

Since $\varphi_{\rm mod}$ is linearly related to the modulation voltage eventually makes the modulation depth $\varphi_{\rm mod}$ jump, the actual reduction is related to the contact resistance size, and the relationship between contact resistance and $\varphi_{\rm mod}$ reduction is shown in Table 1.

Based on this, the simulation calculation of the demodulation coefficients is carried out according to the Bessel function equation in Eq. (15).

(15) $${J_n}({k_{\rm mod}}) = \sum\limits_{k = 0}^\infty {\displaystyle{{{{( - 1)}^{\rm k}}{{\bigg(\displaystyle{{{\varphi _{\rm mod }}} \over 2}\bigg)}^{\rm n + 2k}}} \over {{\rm k}!\left( {n + {\rm k}} \right)!}}}$$

As can be seen above, when the compensation capacitor is falsely connected, the modulation voltage will jump, resulting in a jump in the modulation depth $\varphi_{\rm mod}$. Since *k*_bessel24_ is calculated from the steady-state value of the interference light intensity information, the change in the value of *k*_bessel24_ during the jump in $\varphi_{\rm mod}$ is a monotonic nonlinear change in the modulation interval of the modulation voltage. After the electronic unit detects the deviation of *k*_bessel24_ from the set value, it starts to adjust the modulating voltage *U*_m_. Since the adjustment period of the electronic unit is limited by the modulating frequency *ω* of the modulating voltage, the adjustment has a certain hysteresis effect, so the whole adjustment process is an oscillation stabilization process, which is reflected in the current demodulation algorithm and outputs a non-stable abnormal current.

**Figure 5:**
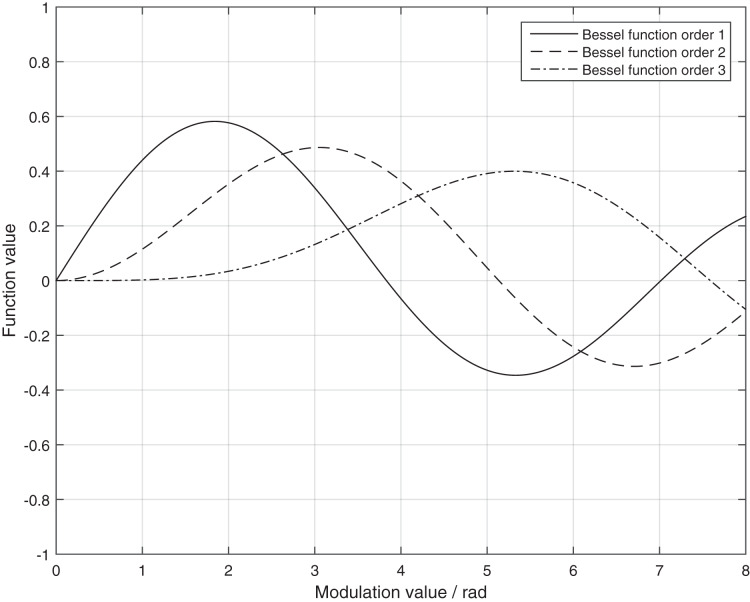
Graph of first-order, second-order and four-order Bessel function. The solid line is the Bessel function order 1. The dashed line is the Bessel function order 2. The dash dot is the Bessel function order 3.

At this time, the modulation voltage change on the modulator is affected by the contact resistance, when the contact resistance is too small modulation voltage change is small, and the original alarm mechanism because of the need to meet the full range of current measurement of the Faraday phase shift of the cosine change, according to Eq. (14) can be issued when the *P*_int2_ drop of 3.4% alarm information. According to the simulation calculation of Fig. 5, when the first-order and second-order Bessel coefficients are equal, $\varphi_{\rm mod}$ is 2.63, and only when $\varphi_{\rm mod}$ is lower than 2.5, the second-order Bessel coefficient can fall by more than 3.4%, thus issuing an alarm message. Table 1 shows that when the contact resistance of the compensation circuit is less than 10 $\Omega$, the modulation depth change is less than 5%, $\varphi_{\rm mod}$ is greater than 2.5 and does not trigger the alarm mechanism of FOCT, but at this time the modulation depth change in phase will be solved to obtain a large error current makes the DC protection device misjudged as a fault within the DC differential protection operation, resulting in accidents.

In order to solve the problem that the sampling value data is abnormal and cannot be alarmed due to the false connection of compensation capacitor, this article proposes an abnormality handling mechanism based on the small change of modulation depth for real-time dynamic current compensation. We simulate the ratio of the first-order Bessel coefficient to the second-order Bessel coefficient *k*_bessel12_ and the ratio of the second-order Bessel coefficient to the fourth-order Bessel coefficient *k*_bessel24_.

According to the simulation results, the ratio of the first-order Bessel coefficient to the second-order Bessel coefficient, *k*_bessel12_, and the ratio of the second-order Bessel coefficient to the fourth-order Bessel coefficient, *k*_bessel24_, vary monotonically when the modulation depth $\varphi_{\rm mod}$ is around the modulation point 2.63. The monotonicity of the values of *k*_bessel24_ and *k*_bessel12_ in the modulation interval can be used to establish a table of the correspondence between *k*_bessel24_ and *k*_bessel12_, so that the tiny working interval of *k*_bessel12_ can be obtained from the value of *k*_bessel24_ by the dynamic table lookup method, and then the linear interpolation can be used to obtain the precise. The exact time value of *k*_bessel12_ is then obtained within the tiny interval using linear interpolation.

(16) $${k_{{\rm bessel12}}} = {y_0} + \displaystyle{{{y_1} - {y_0}} \over {{x_1} - {x_0}}}({k_{{\rm bessel24}}} - {x_0})$$

In Eq. (16), *x*_0_ and *x*_1_ are the starting and final values of *k*_bessel24_ in the tiny working interval, and *y*_0_ and *y*_1_ are the starting and final values corresponding to the *k*_bessel12_ table, respectively, from which the improved current solution equation can be obtained.

(17) $$I \approx \displaystyle{{{k_{{\rm bessel12}}}} \over {4VN}}\displaystyle{{{P_{{\mathop{\rm int}} 1}}} \over {{P_{{\mathop{\rm int}} 2}}}}$$

In order to ensure the accuracy of the dynamic compensation solution, the dynamic adjustment interval of $\varphi_{\rm mod}$ and the correspondence table between *k*_bessel12_ and *k*_bessel24_ within this interval can be set according to the calculation capability and accuracy of the electronic unit in practical engineering. When the value of *k*_bessel24_ deviates from the modulation point, the modulation depth $\varphi_{\rm mod}$ is adjusted by changing the amplitude of the modulation voltage, and the current solution value is compensated dynamically in real time. *k*_bessel24_ continues to restore the solution system to normal by adjusting the modulation voltage when it exceeds the dynamic adjustment interval, at which time dynamic current compensation is no longer possible and an alarm signal is issued.

The above improvement method is only for FOCT in modulation voltage loop abnormalities in the original solution to increase the dynamic compensation coefficient and abnormal alarm information does not change the FOCT transmission principle, and *k*_bessel24_ value only responds to changes in modulation depth. Therefore, this improvement measure can not only improve the real-time sampling accuracy of FOCT when the modulation voltage changes, but also greatly improve the alarm sensitivity of FOCT when the modulation voltage is abnormal. This is a relatively effective solution for FOCTs that have been operating in large numbers at this stage.

## Dc current transformer and dc protection integrated test system

Because the improved FOCT solution adds real-time dynamic current compensation, its calculation accuracy may affect its current compensation accuracy when the current changes rapidly, so the current step response characteristics and frequency response capability should be tested while verifying the effectiveness of the improved measures to test whether the transmission characteristics of FOCT will affect the correctness of DC protection action when the fault occurs (Chen, Xu & Wang, 2020; Li et al., 2020; Jahn, Hohn & Norrga, 2018; Gusarov et al., 2018; Li et al., 2019a). Since the DC protection action depends on the information of control system and a large number of environmental variables, a complete test cannot be performed in the field (Dong, 2022), so this article uses the completed Poyang Lake EHV DC simulation system to build a complete DC test platform including FOCT, DC protection, DC control system and EHV DC simulation system in the laboratory in combination with DC control system. The configuration of the experimental platform is shown in Table 2.

**Table 2:** Experimental platform configuration.

Test item	Fault alarm
RACK	PB5*2
Input and output modules	GTAO*1, GTDO*1
Digital emulation power amplifiers	PA60B*6
Circuit breaker simulation units	PSS02B*1
Power supplies	DC power supplies*1

### Design of the detection system

**Figure 6:**
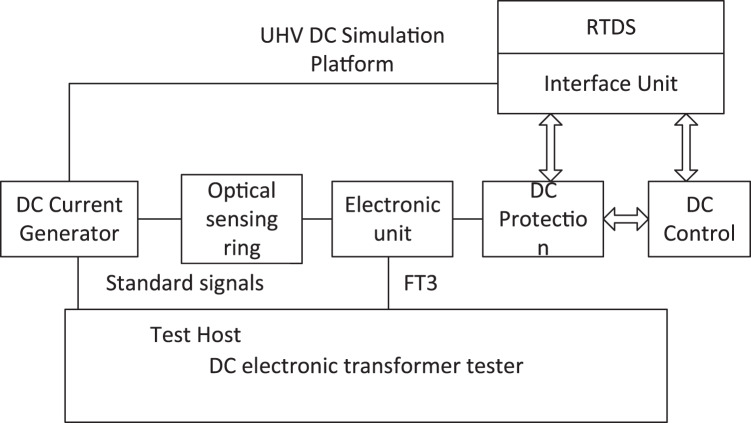
Transient test system for DC current transformer and DC protection.

This article designs an integrated transient test system for DC current transformers and DC protection, as shown in Fig. 6. The testing system is composed of primary current generation module, DC transformer detection module, UHV DC simulation system and DC control system, with optical sensors, electronic units and DC protection as the tested objects.

The EHV simulation platform consists of RTDS simulation system and interface unit. The RTDS simulation system simulates and calculates the EHV DC system, and then sends the data to the DC control and protection system through the interface unit using bi-directional communication, and receives the control information from the control and protection system to complete the closed-loop simulation state. At the same time, the interface unit converts the sampled value information into a small analog voltage signal to control the DC current generator to send out DC current, and changes the sampling value mapping of DC protection to map the sampled value of the low end of pole 2 to the sampling value input of the external full fiber optic current transformer (FOCT) for experiments.

DC current generator with both transient step and steady-state current output capability, but also to have the ability to receive the simulation platform data trigger output, can only be achieved using a linear power amplifier, due to laboratory conditions DC current generator maximum rated current of only 300 A, in order to meet the needs of the experimental maximum current of 5,000 A, so the use of long current wire through the sensor through the heart of 20 turns, the output current will be amplified in the same proportion to meet the needs of the test.

The DC electronic transformer tester synchronously collects the small voltage signal of the built-in standard resistance of DC current generation as the standard source and the FT3 signal of the electronic unit output as the test signal to complete the transient step response and frequency response test of FOCT.

### Test verification

Based on the test system in Fig. 6, offline and online simulation tests were carried out on the improved FOCT and DC protection system respectively. According to the relevant regulations, the transient capability of the improved FOCT is judged by testing parameters such as response time and overshoot under step response and frequency response characteristics, and whether it meets the requirements.

Five tests were carried out at 1,500 A, 600 A and 300 A respectively. As the test product sampling rate was 10 kHz and the sampling interval was 100 μs, the test results were somewhat random, times less than the required 250, 78.2, 108.5 and 89.7 μs respectively, much less than the 250 μs time and 20% overshoot requirement. The average of the test results is shown in Table 3.

**Table 3:** Test data of step response characteristics.

Test item	Rise time (μs)	Response time (μs)	Overshoot (%)	Fault alarm
1,500 A	94.4	77.4	7.6	/
600 A	54.6	87.1	2.9	/
300 A	76.5	83.8	4.1	/

Five tests were conducted at 1.2, 1.5, and 2.5 kHz frequencies, respectively, and the test results were consistent, and the average of the test results is shown in Table 4.

**Table 4:** Test data of frequency response characteristics.

Test item	Ratio error (ε%)	Frequency error (Hz)	Response time (μs)	Fault alarm
1.2 kHz	−0.46	−0.0026	45.6	/
1.5 kHz	−0.67	−0.0057	46.2	/
2.5 kHz	−0.85	−0.0061	45.1	/

From the test results, it can be seen that the overall transient indexes of the FOCT meet the step and frequency response indexes of the DC current transformer after adopting the anomaly handling mechanism based on the small change of modulation depth and real-time dynamic current compensation, and the fault alarm mechanism will not be triggered during the step and frequency response tests.

**Figure 7:**
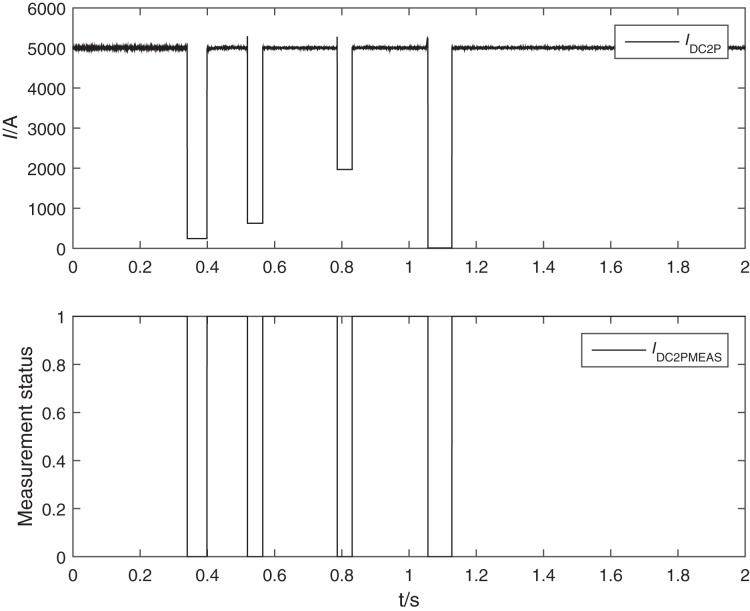
Waveform of FOCT unstable connection test. IDC2P is the Foct current. IDC2PMEAS indicates the FOCT state. If IDC2PMEAS is equal to one, it means that the measurement is abnormal.

Finally, the simulation platform is used to put the DC system all in the running state with load for online simulation test, and the sampling value of the low end of pole 2 of the DC protection is replaced by the actual FOCT. The DC current generator outputs the DC current value according to the work of the simulation system, and the DC protection is in the normal working state. First, simulating the fault of FOCT in the field, the compensation circuit is falsely connected, and after several tests, it can reliably alarm and block the protection when the sampling value output is abnormal in the falsely connected process, and open the protection when the DC current transformer outputs the correct current value, and the DC protection recording is shown in Fig. 7. Second, several fault simulation tests were conducted inside and outside the zone at pole 2, and the action behavior of DC protection was also correct and reliable. That is, the modified FOCT can effectively distinguish the compensation circuit fault from the primary system fault and meet the requirements of DC protection for DC current transformer reliability. It shows that the modified FOCT can effectively distinguish between compensation loop faults and primary system faults, meeting the requirements of DC protection for DC current transformer reliability.

## Conclusion

In this article, we analyze the modulation principle of FOCT modulation loop, demodulation method, abnormal alarm mechanism and the reason of abnormal compensation loop causing DC protection misoperation through simulation calculation, so as to propose the abnormal processing mechanism based on the small change of modulation depth and real-time dynamic current compensation, which effectively solves the problem of The problem of abnormal output current caused by sudden change of modulation voltage in the modulation circuit and the inability to warn is solved effectively.

Finally, through the design of a set of DC current transformer, DC protection integrated transient test system, the actual improved FOCT offline and online transient test, to verify the effectiveness of the improved method and the practicality of the testing system, for the later FOCT engineering application and upgrade and replacement in smart grid to provide a complete set of testing means.

In the future, the next research will be carried out on the diversification of functions and the improvement of detection stability and universality with regard to the anomalous behaviour that occurs when optical DC current transformers are intelligent.

## Supplemental Information

10.7717/peerj-cs.1132/supp-1 Supplemental Information 1 Simulation model of modulation circuit and the bessel function code. The oscilloscope was used to measure modulation voltage. The bessel function code was used to plot Figure 5.

## Additional Information and Declarations

### Data Availability

The following information was supplied regarding data availability: The raw data is available in the Supplemental File which includes the simulation model of modulation circuit and the Bessel function code.
